# Comparing Mental Health During the COVID-19 Lockdown and 6 Months After the Lockdown in Austria: A Longitudinal Study

**DOI:** 10.3389/fpsyt.2021.625973

**Published:** 2021-03-30

**Authors:** Christoph Pieh, Sanja Budimir, Elke Humer, Thomas Probst

**Affiliations:** ^1^Department for Psychotherapy and Biopsychosocial Health, Danube University Krems, Krems, Austria; ^2^Department of Work, Organization and Society, Ghent University, Gent, Belgium

**Keywords:** mental health, COVID-19, depression, anxiety, insomnia, stress

## Abstract

The novel coronavirus disease (COVID-19) has repeatedly been reported to impair mental health. This longitudinal study evaluated mental health at the emergence of the COVID-19 pandemic (t1) and 6 months later (t2) in Austria. Indicators of mental health were depression (PHQ-9), anxiety (GAD-7), sleep quality (ISI), perceived stress (PSS-10), as well as quality of life (WHO-QOL BREF) and well-being (WHO-5). In total, *N* = 437 individuals participated in both surveys (52.9% women). The number of participants with clinically relevant depressive, anxiety, or insomnia symptoms did not differ statistically significantly between t1 and t2 (*p* ≥ 0.48). The prevalence of moderate or severe (clinically relevant) (1) depressive symptoms changed from 18.3% to 19.7% (*p* = 0.48), (2) anxiety symptoms from 16.5 to 15.6% (*p* = 0.67), and insomnia from 14.6 to 15.6% (*p* = 0.69) from t1 to t2. Bonferroni-corrected *t*-tests showed that the stress level (PSS-10) decreased, and well-being (WHO-5) increased. However, effect sizes do not seem to be clinically relevant (Cohen‘s *d* < 0.2). Results suggest that detrimental health consequences of the COVID-19 pandemic persisted several months after its outbreak and the end of the lockdown measures, respectively. Regarding well-being and stress, there is a slight trend toward improvement.

## Introduction

The novel coronavirus disease 2019 (COVID-19) pandemic and the resulting measures to mitigate the uncontrolled spreading of the virus dramatically affect health, economics, and social connections across the world (1). Several recent studies highlight that many psychological problems emerged progressively during this state of public health crisis (2). Several papers report a high prevalence of mental symptoms during the COVID-19 pandemic. For example, a meta-analysis based on 9,074 participants found a prevalence of 34% for depression, 32% for anxiety symptoms, and 30% for stress (3). Fears of infection and possible consequences as serious as death are among the suggested causes for these high rates of mental health symptoms during COVID-19 (4). Furthermore, governmental restrictive measures not only have led to substantial adverse effects on the global economy, causing a strong worldwide increase in the unemployment rate (5), but also have been reported to increase mental health issues like anxiety and depression (6). Reduction of social contact, isolation, and quarantine range among the most important risk factors for psychological distress that individuals are exposed to during the COVID-19 pandemic, that is, during lockdowns (7).

There is a paucity of information about how mental health will evolve during this public health emergency. The little longitudinal research conducted so far points to an increase in mental health issues at the beginning of the COVID-19 pandemic and a decrease in psychological distress thereafter as summarized in the following. A study conducted on college students in China revealed that after 2 weeks of COVID-19 confinement measures (February 2020), an increase in anxiety and depressive symptoms as compared to the time before the confinement measures (December 2019) emerged (8). During the confinement, students were required to stay at home and learn from a distance, as well as to report daily about their health and body temperature to the officers of the school. Therefore, enforced infection-control measures and undergoing confinement measures were suggested to cause negative psychological impacts (8). Another longitudinal study was conducted in the Chinese general population during the initial phase of the COVID-19 outbreak and 4 weeks later during the epidemic. A significant reduction in post-traumatic stress disorder symptoms at the second survey was observed, whereas depression, anxiety, and stress symptoms did not differ (9). During the first survey (end of January 2020 until the beginning of February 2020), the number of confirmed COVID-19 cases and related deaths rapidly increased in China, while the second survey (end of February 2020 until the beginning of March 2020) was conducted after daily newly confirmed COVID-19 cases had rapidly decreased (10). The authors concluded that the rapid measures imposed by the Chinese government to combat the spreading of the virus were instrumental not only in reducing the uncontrolled spreading of the virus but also in preventing a higher psychological impact of COVID-19, as prolonged lockdown measures dramatically affect mental health, especially in the youth (9). A longitudinal US study observed a sharp increase in psychological distress in the initial stages of the COVID-19 outbreak in the US as the COVID-19 crisis evolved (March–April 2020) and lockdown measures were initiated. Interestingly, the substantial increase in psychological distress levels largely diminished several months after the COVID-19 outbreak (June 2020) (11). A further longitudinal population-based study investigated mental health issues before and several times throughout the COVID-19 pandemic in the UK (12). A strong increase in mental health problems emerged during the COVID-19 lockdown (April 2020) as compared to pre-pandemic data (2017–2019). The high levels of mental health problems declined between April and June 2020, the time where daily newly confirmed COVID-19 cases declined as well as lockdown measures were eased (10); however, despite the decline, mental health problems remained elevated compared to pre-pandemic levels (12). Taken together, these studies point to a reduction of mental health issues with the prolongation of the COVID-19 pandemic—at least to a certain extent. However, it is not possible to draw causal conclusions about the underlying reasons, such as easing of lockdown measures or a decrease in the perceived infection risk due to a lower number of daily confirmed COVID-19 cases. Furthermore, there is a paucity of longitudinal studies that have examined how mental health changed on a long-term basis within the same sample of participants.

In Austria, the first COVID-19 cases were confirmed on the 25^th^ of February 2020. The Austrian government introduced obligatory COVID-19 lockdown measures on the 16^th^ of March 2020, which lasted until April 30^th^. A nationwide curfew entailed restrictions in movement and activities with several exceptions. These exceptions included addressing immediate danger, meeting basic needs, fulfilling work responsibilities, assistance for people in need, and outdoor activities only with the people from the same household, with at least 1-m distance between people. The easing of the measures started on the 14^th^ of April 2020 and included reopening of shops beyond basic services with the obligation of wearing masks in public transport and all shops. Daily confirmed cases peaked in Austria during the COVID-19 lockdown with >1,000 confirmed cases per day at the end of March. With the end of the lockdown, daily cases decreased and remained at a low level (<100 cases/day) until the end of June 2020. The decrease in the number of confirmed COVID-19 cases after the lockdown was accompanied by decreased movement, controlled by allowing travel only to countries with the lowest number of COVID-19 cases. From July to September 2020, daily cases started to increase again (13).

We recently reported a major increase in mental health problems, including depressive, anxiety, and insomnia symptoms, in the Austrian general population during the COVID-19 lockdown compared to studies conducted before the COVID-19 pandemic (14). In more detail, during the COVID-19 lockdown, depressive symptoms (21%) and anxiety symptoms (19%) were higher than previous epidemiological data [6% for depression in an Austrian sample in 2019 (15) and 6% for anxiety in studies from Germany in 2017 (16)]. Moreover, we compared mental health between lockdown in April 2020 vs. after lockdown in June 2020 in Austria, and we did not find the positive changes (17) as in the studies cited above, but, in fact, found a higher number of new onsets of depression than remissions of depression (18). In general, previous cross-sectional (19–22) as well as longitudinal studies (23, 24) investigating psychological outcomes for subjects who have been quarantined due to global infections compared to those not quarantined reveal a higher prevalence of psychological symptoms, such as depression, insomnia, and anxiety, which often sustain even in the long-term period. However, evidence also exists that the COVID-19 outbreak affects mental health in the general population independent of lockdown measures, likely due to fears related to infections, pervasive anxiety, frustration and boredom, as well as loneliness (25–28).

Therefore, research is needed to reveal whether mental health symptoms related to the COVID-19 pandemic persist in the long term or rather decline after the lockdown measures end. As such, this longitudinal study aimed to evaluate if mental health problems declined in the Austrian general population 6 months after the COVID-19 outbreak as compared to the first months of the COVID-19 outbreak when lockdown measures were in place. We hypothesized that mental health improved at 6 months after the lockdown as compared to the time during the lockdown measures.

## Materials and Methods

### Study Design

This longitudinal study comprises two online surveys, which were performed during (t1) and after (t2) the COVID-19 lockdown through Qualtrics® (29). The first survey started after 4 weeks of lockdown in Austria (10^th^ of April 2020) and ended with the end of the nationwide curfew on the 30^th^ of April 2020. The second survey (t2) was conducted 6 months after the start of the lockdown in Austria (from the 7^th^ to 21^st^ of September 2020).

### Study Sample

In April 2020 (t1), a representative study sample according to age, gender, education, and region for Austria was recruited through Qualtrics® (29). The sociodemographic characteristics of the representative sample of 1,005 participants evaluated during the lockdown (t1) have been reported in detail previously (13). The same participants were re-contacted in September (t2), and N=437 (response rate of 43.5%) participated again in the study. The characteristics of participants in both surveys (i.e., the study sample) are presented in Table 1.

**Table 1:** Study sample characteristics (*N* = 437).

**Variable**	**Frequencies (percent)**
**Gender**	
Women	231 (52.9)
Men	206 (47.1)
**Age**	
18–24	26 (5.9)
25–34	49 (11.2)
35–44	68 (15.6)
45–54	99 (22.7)
55–64	100 (22.9)
65+	95 (21.7)
**Region**	
Burgenland	16 (3.7)
Lower Austria	83 (19.0)
Vienna	102 (22.3)
Carinthia	27 (6.2)
Styria	55 (12.6)
Upper Austria	82 (18.8)
Salzburg	35 (8.0)
Tyrol	27 (6.2)
Vorarlberg	10 (2.3)
**Education**	
No school education	1 (0.2)
Secondary school	11 (2.5)
Apprenticeship	153 (35.0)
High school	109 (24.9)
University	163 (37.3)

### Measures

#### Depressive Symptoms (PHQ-9)

Depressive symptoms were measured with the PHQ-9, which is a depression module of the Patient Health Questionnaire (30). It measures depressive symptoms with nine self-rating items on a four-point scale, ranging from 0 to 3 (maximum score of 27). Cut-off points are 5 for mild depression, 10 for moderate depression, and at least 15 for severe levels of depression (31). The 10-point cut-off was used in the present study to define clinically relevant depression. Cronbach's alpha for the PHQ-9 for the first measuring point was α =.90, and it was α =.92 for the second measuring point.

#### Anxiety (GAD-7)

Anxiety symptoms were measured with the validated instrument Generalized Anxiety Disorder 7 scale (32, 33) (GAD-7). It measures anxiety with seven self-rating items on a four-point scale, from 0 to 3 (maximum score of 21). Cut-off points are 5 for mild, 10 for moderate, and 15 for severe anxiety symptom levels. The 10-point cut-off was used in the current study to define clinically relevant anxiety. Cronbach's alpha for anxiety for the first measuring point was α = 0.91 and was α = 0.92 for the second measuring point.

#### Insomnia (ISI)

Problems with sleeping in the form of insomnia were measured with a validated questionnaire: the Insomnia Severity Index (ISI) (34). It measures sleep quality and insomnia on seven self-reported items on a four-point scale ranging from 0 to 4 (maximum score of 28). Total scores below 7 indicate no clinically significant insomnia, scores of between 8 and 14 indicate subthreshold insomnia, scores of between 15 and 21 points indicate clinical insomnia (moderate severity), and scores of 22–28 points indicate severe clinical insomnia. In this study, 15 points were used as the cut-off to define clinically relevant insomnia. Cronbach's alpha for insomnia for the first measuring point was α = 0.86 and was α = 0.88 for the second measuring point.

#### Perceived Stress (PSS-10)

The perceived stress level was measured with a reliable and valid measure of stress level over the previous month, PSS-10 (35). It is measured with 10 items on a five-point scale ranging from 0 to 4 (maximum score of 40) with a higher score indicating higher perceived stress. Cronbach's alpha for PSS-10 was α = 0.90 for both measuring points.

#### Quality of Life (WHO-QOL BREF)

Quality of life was measured with a reliable, validated (36), 26-item self-rating instrument, WHOQOL-BREF (37). It measures physical health, psychological health, social relationships, and the environment in the period of the previous past 2 weeks. In this study, only the psychological domain was used as an indicator of the mental quality of life. It is estimated on six items on a Likert scale ranging from 1 to 5, with higher scores indicating higher psychological health. The general population norm for the WHOQOL-BREF psychological domain has been reported to be 70.6 (SD = 14.0) (38). Cronbach's alpha for the psychological domain for the first measuring point was α = 0.87 and was α = 0.89 for the second measuring point.

#### Well-Being (WHO-5)

Well-being was measured with the WHO-5 questionnaire (39), which has good psychometric properties (40, 41). It measures well-being with five self-rating items rated on six-point Likert scales ranging from 1 to 5 (maximum score of 25) with a higher score indicating higher well-being. Cronbach's alpha for WHO-5 for the first measuring point was α = 0.92 and was α = 0.93 for the second measuring point.

### Statistical Analysis

Data were analyzed in SPSS version 24 (IBM Corp, Armonk, NY, USA). Descriptive statistics were conducted to describe the demographic characteristics and scales mean values.

Differences between two measurement points were evaluated by *t*-test pairwise comparisons, and Bonferroni correction for multiple comparisons was applied for results interpretation, considering *p* < 0.008 as significant (*p* < 0.05/6 *t*-tests). Potential differences in outcome measures during the COVID-19 lockdown (t1) between responders vs. non-responders were assessed by independent *t*-tests. The *t*-tests performed were two-tailed, and the Bonferroni-corrected significance was set to *p* < 0.008 (*p* < 0.05/6 *t*-tests). As the effect size measure, Cohen's d was calculated, which can be interpreted as follows: small effect 0.2–0.5, medium effect 0.5–0.8, and large effect > 0.8.

McNemar chi-squared tests were performed to investigate differences between symptom severity categories between the two time points, and Bonferroni correction for multiple comparisons was applied for results interpretation, considering *p* < 0.016 as significant (*p* < 0.05/3 tests). Chi-squared tests were conducted to investigate differences in symptom severity categories between responders vs. non-responders during the COVID-19 lockdown (t1). Bonferroni correction for multiple comparisons was applied for results interpretation, considering *p* < 0.016 as significant (*p* < 0.05/3 tests).

### Ethical Consideration

This study was conducted following the Declaration of Helsinki and approved by the Ethics Committee of the Danube University Krems, Austria (ethical number: EK GZ 26/2018-2021). All participants gave electronic informed consent for participation and completing the questionnaires. Data were collected anonymously without IP addresses or GPS tracking, and this procedure was approved by the data protection officer of the Danube University Krems.

## Results

Results for mean scores (M) and standard deviations (SD) for PHQ-9, GAD-7, ISI, PSS-10, WHO-QOL BREF, and WHO-5, between the first and second measuring points, are presented in Table 2.

**Table 2:** Measures of psychological health, well-being, perceived stress, depression, anxiety, and insomnia during the lockdown (t1) as compared to 6 months after the initiation of the lockdown (t2) in *n* = 437 individuals.

		**t1**	**t2**	**Statistics**
PHQ-9	M	5.74	5.51	t(436) = 1.37; p = 0.172; d = 0.07
	SD	5.50	5.65	
GAD-7	M	5.35	4.97	t(436) = 2.24; p = 0.026; d = 0.11
	SD	4.69	4.82	
ISI	M	7.97	7.84	t(436) = 0.61; p = 0.539; d =0.03
	SD	5.83	5.98	
PSS-10	M	15.27	14.25	t(436) = 3.90; p < 0.001; d=0.19
	SD	7.97	7.93	
WHOQOL BREF	M	70.21	71.50	t(436) = −2.11; p = 0.036; d =0.10
(psychological domain)	SD	19.35	20.28	
WHO-5	M	15.39	15.97	t(436) = −2.85; p = 0.005; d =0.14
	SD	5.57	5.77	

*P, p-values (2-tailed); M, mean score; SD, standard deviation, t, t-test; PHQ-9, patient health questionnaire 9 scale; GAD-7, generalized anxiety disorder 7 scale; ISI, insomnia severity index; PSS-10, perceived stress scale 10; WHO-QOL BREF, quality of life questionnaire of the World Health Organization (WHO); WHO-5, Well-being questionnaire of the World Health Organization (WHO)*.

Mean values on each scale in the period after the lockdown are showing slight improvement compared to the values measured during the lockdown. According to pairwise comparisons with *t*-tests, statistically significant differences (*p* ≤ 0.005) were observed only for perceived stress (PSS-10) and well-being (WHO-5), indicating a decrease in perceived stress (M_t1_ = 15.27, SD_t1_ = 7.97, M_t2_ = 14.25, SD_t2_ = 7.93) and an increase in well-being (M_t1_ = 15.39, SD_t1_ = 5.57, M_t2_ = 15.97, SD_t2_ = 5.77) after the lockdown. However, the effect sizes (Cohen's *d*) for both scales (d_PSS−10_ = 0.19; d_WHO−5_ = 0.14) were very low (below 0.2).

The responders (*n* = 437) did not differ from non-responders (*n* = 568) in any outcome variables during the COVID-19 lockdown (*p* ≥ 0.010) as summarized in Table 3.

**Table 3:** Measures of psychological health, well-being, perceived stress, depression, anxiety, and insomnia during the lockdown (t1) in responders (*n* = 437) compared to non-responders (*n* = 568).

		**Responders**	**Nonresponders**	**Statistics**
PHQ-9	M	5.74	6.53	t(1003) = −2.29; p = 0.022; d = 0.15
	SD	5.50	5.31	
GAD-7	M	5.35	6.22	t(1003) = −2.92; p = 0.04; d = 0.19
	SD	4.69	4.67	
ISI	M	7.97	8.57	t(1003) = −1.65; p = 0.099; d = 0.11
	SD	5.83	5.59	
PSS-10	M	15.27	16.51	t(1003) = −2.63; p = 0.010; d = 0.17
	SD	7.97	7.01	
WHOQOL BREF	M	70.21	69.54	t(1003) = 1.74; p = 0.082; d = −0.04
(psychological domain)	SD	19.35	18.20	
WHO-5	M	15.39	14.79	t(1003) = 0.57; p = 0.571; d = −0.11
	SD	5.57	5.26	

*p: p-values (2-tailed); M: mean score; SD: standard deviation, t: t-test; PHQ-9: Patient Health Questionnaire 9 scale; GAD-7: Generalized Anxiety Disorder 7 scale; ISI: Insomnia Severity Index; PSS-10: Perceived Stress Scale 10; WHO-QOL BREF: Quality of Life questionnaire of the World Health Organization (WHO); WHO-5: Well-being questionnaire of the World Health Organization (WHO)*.

Comparisons of the number of participants below/above the cut-off scores for moderate (i.e., clinically relevant) depression/anxiety/insomnia are given in Table 4.

**Table 4:** Number of participants exceeding the cut-off score for moderate depression/anxiety/insomnia measured during the lockdown (t1) and 6 months after the initiation of the lockdown (t2).

						**McNemar Test**	**N**
			**PHQ9_t1**				
			<10	≥10	Total		
**PHQ9_t2**	<10	f(%)	329 (75.3)	22 (5)	351 (80.3)	0.480^a^	437
	≥10	f(%)	28 (6.4)	58 (13.3)	86 (19.7)		
	Total	f(%)	357 (81.7)	80 (18.3)	437 (100)		
			**GAD7_t1**				
			<10	≥10	Total		
**GAD7_t2**	<10	f(%)	342 (78.3)	27 (6.2)	369 (84.4)	0.672	437
	≥10	f(%)	23(5.3)	45 (10.3)	68 (15.6)		
	Total	f(%)	365 (83.5)	72 (16.5)	437 (100)		
			**ISI_t1**				
			<15	≥15	Total		
**ISI_t2**	<15	f(%)	342 (78.3)	27 (6.2)	369 (84.4)	0.694	437
	≥15	f(%)	31(7.1)	37 (8.5)	68 (15.6)		
	Total	f(%)	373 (85.4)	64 (14.6)	437 (100)		

*PHQ-9: Patient Health Questionnaire 9 scale; GAD-7: Generalized Anxiety Disorder 7 scale; ISI: Insomnia Severity Index*.

There was no significant difference in any measure of mental health between t1 and t2. At t1 (during the lockdown), 5% of the total sample were above the clinical cut-off for depression (≥10 points) and became non-clinical at t2 (6 months after the lockdown), while 6.4% initially were below the cut-off but above the cut-off for clinical depression 6 months after the lockdown (*p* = 0.48). In total, *n* = 80 (18.3%) scored above the PHQ-9 cut-off (≥10 points) for moderate depressive symptoms at t1 and *n* = 86 (19.7%) at t2.

For anxiety, 6.2% of the total sample were above the clinical cut-off (GAD-7 score ≥ 10 points) at t1 and became non-clinical at t2, while 5.3% initially were below the cut-off at t1 but above the cut-off at t2 (*p* = 0.67). In total, *n* = 72 (16.5%) scored above the GAD-7 cut-off (≥ 10 points) for moderate anxiety symptoms at t1 and *n* = 68 (15.6%) at t2.

For insomnia, 6.2% of the total sample were above the clinical cut-off (ISI score ≥ 15 points) at t1 and became non-clinical at t2, while 7.1% were initially below the cut-off but above the cut-off for clinical insomnia at t2 (*p* = 0.69). In total *n* = 64 (14.6%) scored above the ISI cut-off (greater-equal 15 points) for moderately clinical insomnia at t1 and *n* = 68 (15.6%) at t2.

These findings indicate that there is no clinically relevant change in mental health between the time during the COVID-19 lockdown and 6 months after the lockdown.

The responders (*n* = 437) did not differ from non-responders (*n* = 568) in any measure of mental health during the COVID-19 lockdown (*p* ≥ 0.066) as summarized in Table 5.

**Table 5:** Number of participants exceeding the cut-off score for moderate depression/anxiety/insomnia measured during the lockdown (t1) in responders (*n* = 437) compared to nonresponders (*n* = 568).

		**Responders**	**Non-responders**	**Total**	**Statistics**
PHQ9_t1 f(%)	<10	357 (81.7)	437 (76.9)	794 (79.0)	χ^2^ (1) = 3.37; p = 0.066
	≥10	80 (18.3)	131 (23.1)	211 (21.0)	
GAD7_t1 f(%)	<10	365 (83.5)	449 (79.0)	814 (81.0)	χ^2^ (1) = 3.21; p = 0.073
	≥10	72 (16.5)	119 (21.0)	191 (19.0)	
ISI_t1 f(%)	<15	373 (85.4)	474 (83.5)	847 (84.3)	χ^2^ (1) = 0.676; p = 0.411
	≥15	64 (14.6)	94 (16.5)	158 (15.7)	

*PHQ-9, patient health questionnaire 9 scale; GAD-7, generalized anxiety disorder 7 scale; ISI, insomnia severity index*.

## Discussion

The current study explored mental health 6 months after the start of the COVID-19 lockdown in Austria as compared to the time during the COVID-19 lockdown. The major finding of this study is that the COVID-19 pandemic caused long-lasting detrimental effects on mental health. The number of participants with moderate or severe depressive, anxiety, or insomnia symptoms remained largely unchanged throughout the 6 months. Still, 20% suffer from moderate or severe depressive symptoms, 16% from moderate or severe anxiety symptoms, and 16% from moderate or severe clinical insomnia. Although a statistically significant improvement in perceived stress and well-being 6 months after the lockdown was observed, the effect sizes were very small, indicating no clinically relevant improvement. The results for stress and well-being are in line with our hypothesis, but the hypothesis that mental health improved 6 months after the start of the lockdown must be rejected for the other examined indicators of mental health. Moreover, we could not replicate the result that we found when we compared depression in April vs. June 2020, that is, there were more new onsets of depression than remissions in April vs. June 2020 (18), but not in April vs. September 2020. One explanation for this could be that although the same sample was contacted in June and September, different people responded, and there is only a partial overlap of the sample examined in June (18) and September (current manuscript).

As the current COVID-19 pandemic is a unique event in the recent past, prediction of the development of mental health is only possible to a limited extent. It remains unclear if the experience of previous pandemics or catastrophes allows a forecast of the development. In general, research on mental health consequences due to pandemics does not allow differentiation between negative consequences of the disease itself (such as negative impacts due to the fear of infection) or consequences due to job loss, financial losses, or lockdown measures. In general, previous research demonstrates that large-scale catastrophes, including natural, environmental, or traumatic disasters, are commonly accompanied by a broad range of mental and behavioral disorders (7). Research suggests also that mental health issues can occur immediately after large-scale disasters and then persist for a long time (7).

Previous longitudinal studies conducted during the COVID-19 pandemic suggest that mental health has been substantially affected both by the pandemic itself and the lockdown measures (8, 12). A study conducted in the US observed a relatively quick psychological adaption to the COVID-19 pandemic, showing almost pre-pandemic values several months after the COVID-19 outbreak in the US (11). In contrast to these findings, our study suggests a substantial persistence in mental health problems in Austria, which remained mainly unchanged although severe restrictions were lifted several months before. Although some degree of adjustment and coping after the initial stress of the pandemic was observed—as indicated by a decreased perceived stress level and an increase in well-being—the proportion of participants with mental health problems did not decrease and remained higher than pre-COVID-19 levels, which reported, for example, 4% of the Austrian population being above the cut-off for depression (42).

There might be a constellation of factors that contribute to the long-lasting mental health effects during the COVID-19 pandemic. One possible explanation for the current findings is that the lockdown measures are not primarily causal for the high rates of mental health symptoms. Another explanation would be that mental health symptoms disappear more slowly than they emerged. Thus, it can also be speculated that the lockdown caused long-standing detrimental mental health consequences. Finally, it is also possible that the perceived risk of infection and worries about the health of others contributed to the finding that mental health symptoms persisted at a high level even 6 months after lockdown measures were initiated. In line with this argument, the officially reported daily confirmed COVID-19 cases were even higher at T2 (on average 639 cases per day) as compared to T1 (on average 114 cases per day), as the number of confirmed COVID-19 cases rapidly increased again in September 2020 in Austria after they had persisted at a low level for several months (10).

The generalizability of this study is limited due to the small sample size and a moderate response rate. Moreover, a third measurement point before the COVID-19 lockdown would have been more appropriate to draw causal conclusions about the effect of the COVID-19 lockdown *per se*. A further limitation is that mental health was assessed with self-ratings and not with clinician-based assessments. As people are often biased when they report on their own experiences (43), clinician-rated outcomes might have been better suited to address mental health.

The present findings indicate that mental health problems persisted at a high level several months after the COVID-19 outbreak in Austria. Given the high number of people experiencing depressive, anxiety, and insomnia symptoms, it is essential to ensure adequate mental health care and support during and after the COVID-19 pandemic to those at risk. As mental health problems are associated with a substantial societal and economic burden (44), mitigating measures to address mental health issues during and in the aftermath of the COVID-19 crisis are crucial not only to support mental health but also to reduce healthcare costs due to prolonged recovery times.

## Data Availability Statement

The raw data supporting the conclusions of this article will be made available by the authors, without undue reservation.

## Ethics Statement

The studies involving human participants were reviewed and approved by Ethics Committee of the Danube University Krems, Austria (Ethical number: EK GZ 26/2018-2021). The patients/participants provided their written informed consent to participate in this study.

## Author Contributions

CP, SB, and TP: conceptualization and methodology. CP: formal analysis. CP and SB: investigation. SB: data curation. SB and EH: writing—original draft preparation. TP and CP: writing—review and editing. All authors have read and agreed to the published version of the manuscript.

## Conflict of Interest

The authors declare that the research was conducted in the absence of any commercial or financial relationships that could be construed as a potential conflict of interest.
